# Biocompatible Coating of Encapsulated Cells Using Ionotropic Gelation

**DOI:** 10.1371/journal.pone.0073498

**Published:** 2013-09-09

**Authors:** Friederike Ehrhart, Esther Mettler, Thomas Böse, Matthias Max Weber, Julio Alberto Vásquez, Heiko Zimmermann

**Affiliations:** 1 Biophysik und Kryotechnologie, Fraunhofer IBMT, Sankt Ingbert, Germany; 2 Schwerpunkt Endokrinologie und Stoffwechselerkrankungen, Universitätsmedizin Mainz, Mainz, Germany; 3 Departmento Biologia Marina, Universidad Católica del Norte, Coquimbo, Chile; 4 Lehrstuhl für Molekulare und Zelluläre Biotechnologie/Nanotechnologie, Universität des Saarlandes, Saarbrücken, Germany; Instituto de Engenharia Biomédica, University of Porto, Portugal

## Abstract

The technique of immunoisolated transplantation has seen in the last twenty years improvements in biocompatibility, long term stability and methods for avoidance of fibrosis in alginate capsules. However, two major problems are not yet solved: living cellular material that is not centered in the capsule is not properly protected from the hosts’ immune system and the total transplant volume needs to be reduced. To solve these problems, we present a method for applying fully biocompatible alginate multilayers to a barium-alginate core without the use of polycations. We report on the factors that influence layer formation and stability and can therefore provide data for full adjustability of the additional layer. Although known for yeast and plant cells, this technique has not previously been demonstrated with mammalian cells or ultra-high viscous alginates. Viability of murine insulinoma cells was investigated by live-dead staining and live cell imaging, for murine Langerhans’ islets viability and insulin secretion have been measured. No hampering effects of the second alginate layer were found. This multi-layer technique therefore has great potential for clinical and *in vitro* use and is likely to be central in alginate matrix based immunoisolated cell therapy.

## Introduction

Encapsulation of cells has a long history in biotechnology especially for the immunoisolated transplantation of endocrine cells or tissues. The encapsulation and implantation of Langerhans’ islets with alginate for diabetes therapy is possibly the most famous example for this technique [1-5]. Alginate is the preferred material for this application because it is biocompatible, polymerizes under gentle conditions and is non toxic in both the polymerized state and in solution. It is an unbranched polymer containing manuronic- (M) and guluronic (G) acid. The ionotropic gel is liquid only if monovalent cations are available to saturate the hydroxyl groups of M or G. Alginate solution is viscous but can be used to suspend cells. Capsule formation occurs when droplets of liquid alginate fall into a polymerization bath containing divalent cations, whereas different methods and techniques for droplet formation are yet established [3,6]. Ca^2+^ or Ba^2+^ cross link the alginate chains and form a stable hydrogel. Other di- or multivalent cations form rather weak gels which cannot be used for immobilization purposes [7-8]. Hydrogels made of UHV (ultra high viscous) alginates, characterized by extraordinary high molecular weight, have special properties, which makes them ideal for biomedical applications especially in the field of long term immunoisolation of transplanted cells [4,9,10]. The high molecular weight (high viscosity) alginates are intrinsically more biocompatible than those of low molecular weight (low viscosity) [11]. UHV alginate sheets can be very stable and under special conditions withstand pressures of 2 bar [12].

A broad variety of cell and tissue types have been successfully used for encapsulation including genetically engineered cells, stem cells and stem cell derived surrogate tissue [5,13]. Nevertheless, there are some problems, which hamper the immediate start of clinical trials. Long term stability and fibrosis of the capsule is probably the most commonly addressed problem which is dealt in many publications and shall not be a point of discussion in this work. Here, we consider two other issues: firstly, embedded living material in decentralized position, which is not properly protected against the hosts’ immune system because of too thin alginate layer and secondly, the total transplant volume.

The first issue is a question of transplant safety and long term stability. Donor cells at the capsule’s surface are in danger from the host’s immune system and an overacting or long-term immune reaction can cause other problems like local inflammation and graft failure. Reduced life time of the transplant, the need for repeated transplantations and other medical problems could outweigh the benefits of immunoisolated transplantation. The second issue, the transplant volume, depends mainly on the amount of tissue which is necessary to restore functionality. The ratio of cell/matrix material is usually quite low to avoid cells at the capsules’ surface resulting in a quite high transplantation volume. E.g. to achieve normal blood glucose regulation in a human adult metabolism, about 1 x 10^6^ Langerhans’ islets are necessary. If every islet is singly encapsulated in a 500 µm capsule the total transplantation volume would be about 100 ml. Putting more than one islet in a capsule would increase the risk of an islet, to be placed at the capsule’s surface with the above described risks.

Coated capsules offer a solution to these problems. If a defined layer protects all embedded cells and tissues, higher cell loads are possible, reducing the total transplantation volume. By exploiting the polyanionic character of alginate, polycations are used in alternation with alginate (or another polyanions) to build up a poly-electrolyte shell which increases the stability and protection properties of the capsule [14-16]. Poly-L-Lysine (PLL) is the most popular polycation for this purpose [17-18] but other substances like polyacrylamide (PAA), pectin and chitosan can be used as well for coating a cell loaded alginate core [19-20]. However, PLL is highly immunogenic and has been shown to induce fibrotic overgrowth after implantation in animal models [21]. A final layer of alginate is necessary to mask the PLL layer (so called alginate-PLL-alginate (APA) capsules). Such APA capsules are still more immunogenic than massive alginate capsules, leading to rapid graft failure as the fibrotic overgrowth cuts off the nutrient supply [21-25]. Using sensitive methods like time-of-flight secondary ion mass spectrometry (ToF - SIMS), XPS (X-ray photoelectron spectroscopy) and ATR-FTIR (attenuated total reflection Fourier transform infrared) spectroscopy, recent studies showed that the PLL coating was seldom completely masked by the outer alginate layer and therefore led unavoidably to fibrotic overgrowth and the activation of the hosts immune system [26-28]. Pure alginate capsules without PLL but with a liquid PEG core were presented by Koyama et al. [29] but these capsules suffered from PEG leakage and a massive osmotic driven water flow followed by capsule burst. A different technique is the production of double layered capsules and fibers before gelling [30].

In this work, we present a coating technique for massive UHV alginate capsules based on the publication of Vorlop et al. 1987 [31] who developed an encapsulation technique for producing coated Ca-alginate beads and fibers without the use of polycations. This method was transferred without essential change to a clinically relevant set-up with UHV alginate, pre-crosslinked with the crystal gun (CG) and crosslinked with Ba^2+^ (which produces long-term stable capsules) [9,32]. The aims were to successfully coat core capsules (with and without cells) with biocompatible alginate; to investigate the effect of production parameters on layer thickness and to study layer stability by watching swelling properties in time lapse microscopy. The chosen cell model for the study of viability and proliferation of embedded cells was Rin-m, an insulinoma cell line [33], for viability and functionality rat Langerhans’ islets.

## Materials and Methods

### Cells and cell culture

Rin-m cells were obtained from DSMZ (Braunschweig, Germany) and cultured in RPMI 1640 supplemented with 10% FBS (fetal bovine serum) and Gentamycin. Cell culture media were obtained from Gibco (Invitrogen, Darmstadt, Germany). Spheroids were produced using hanging droplet or free spheroid technique as described elsewhere [34]. Rin-m cells and Langerhans’ islets were stained using fluorescein diacetate (FDA) and ethidium bromide [34] for viability and membrane integrity assessment.

### Islet Isolation and Assessment of Insulin secretion

Langerhans islets were isolated from healthy Wistar rats by a collagenase digestion method [35]. Wistar rats, bred at the University Medical Center, Mainz, 12–16 weeks old were used in all experiments. They were fed a standard pellet diet (B&K) and tap water ad libitum. Before islet isolation rats were euthanized with isoflurane. All animal procedures were approved by our institution’s Ethics Committee (Landesuntersuchungsamt Rheinland-Pfalz) and carried out under license (no. G 12-1-068), in accordance with the Ethical Committee for Animal Research at the University Medical Center, Mainz, Germany. After purification, islets were recovered overnight at 37°C and 5% CO_2_ in TCM 199 (Sigma Aldrich, Steinheim, Germany) supplemented with 1% Penicillin / Streptomycin (Invitrogen, Carlsbad, CA, USA) and 10% fetal calf serum (Seromed Biochrom KG, Berlin, Germany). Single cells and small cell aggregates were removed from the islet suspension by filtration through a 40µm Nylon Cell Strainer (BD Falcon, Franklin Lakes, NJ, USA). The volume of isolated islet was expressed as the number of islet equivalents (IEq), defined as an islet 150 µm in diameter. The Ricordi algorithm was used to convert islet number into IEq [36]. Functional capacity of isolated islets was established by static glucose stimulation assay. Islets were incubated with either 5.5 mM or 16.6 mM glucose in TCM 199 for one hour at 37°C. Supernatant of each sample was collected and stored at -18°C. The insulin content was measured by enzyme-linked immunosorbent assay (High Range Rat Insulin ELISA, DRG, Marburg, Germany). Glucose-stimulated insulin secretion was expressed as stimulation index (SI) and calculated as the ratio of insulin released during exposure to high glucose over the insulin released during low glucose incubation (5.5 mM and 16.6 mM, respectively). Statistical analyses were performed using SPSS 12.0 (SPSS Inc., Chicago, IL, USA). Significant differences were identified using Levene’s Test for Equality of Variances and independent samples t-test. In all tests, the significance level was set at 5% (P < 0.05).

### Alginate preparation

UHV alginates are defined as alginates with viscosities over 30 mPas in a 0.1% solution in aqua bidest. Typical average molecular weights are for *L* *. nigrescens* (LN) 3.3 x 10^5^ to 2.9 x 10^6^, for *L* *. trabeculata* (LT) 1.4 x 10^5^ to 2.6 x 10^6^ g/mol [10]. Ultra high viscous (UHV) alginate was prepared from LN and LT stipes as described elsewhere [1]. A 1:1 mixture of LN and LT alginate was prepared after separate dissolving in sterile 0.9% NaCl solution. Alginate concentrations between 0.1-0.7% (w/v) were used for these experiments. Commercially available low viscosity alginate was obtained from Sigma-Aldrich (Schellendorf, Germany) and used in a concentration of 1% or 2% (w/v).

### Capsule preparation

Alginate capsules were produced using a coaxial air stream device including crystal gun (CG) as described elsewhere [1,32]. Shortly, 1 ml alginate, with or without about 1000 Rin-m spheroids was taken up in a 1 ml syringe. For encapsulation of Langerhans’ islets about 3000 IEq (islet equivalents) were taken up in 500 µl alginate solution. The coaxial air stream encapsulation machine produces tiny alginate droplets, which fall into a polymerization bath containing Ba^2+^ (20 mM BaCl_2_, 5 mM Histidine, 115 mM NaCl, 290-300 mOsmol, pH = 7.3-7.4). If the CG was used, alginate droplets were sprayed with tiny BaCl_2_ crystals before falling into the polymerization bath. Using the CG produces more homogeneously cross-linked alginate beads [1]. Alginate concentration of this capsule „core” was always 0.65%. After 15 min incubation time capsules were harvested and alginate layer application started as follows.

### Application of alginate layers

**Figure 1:**
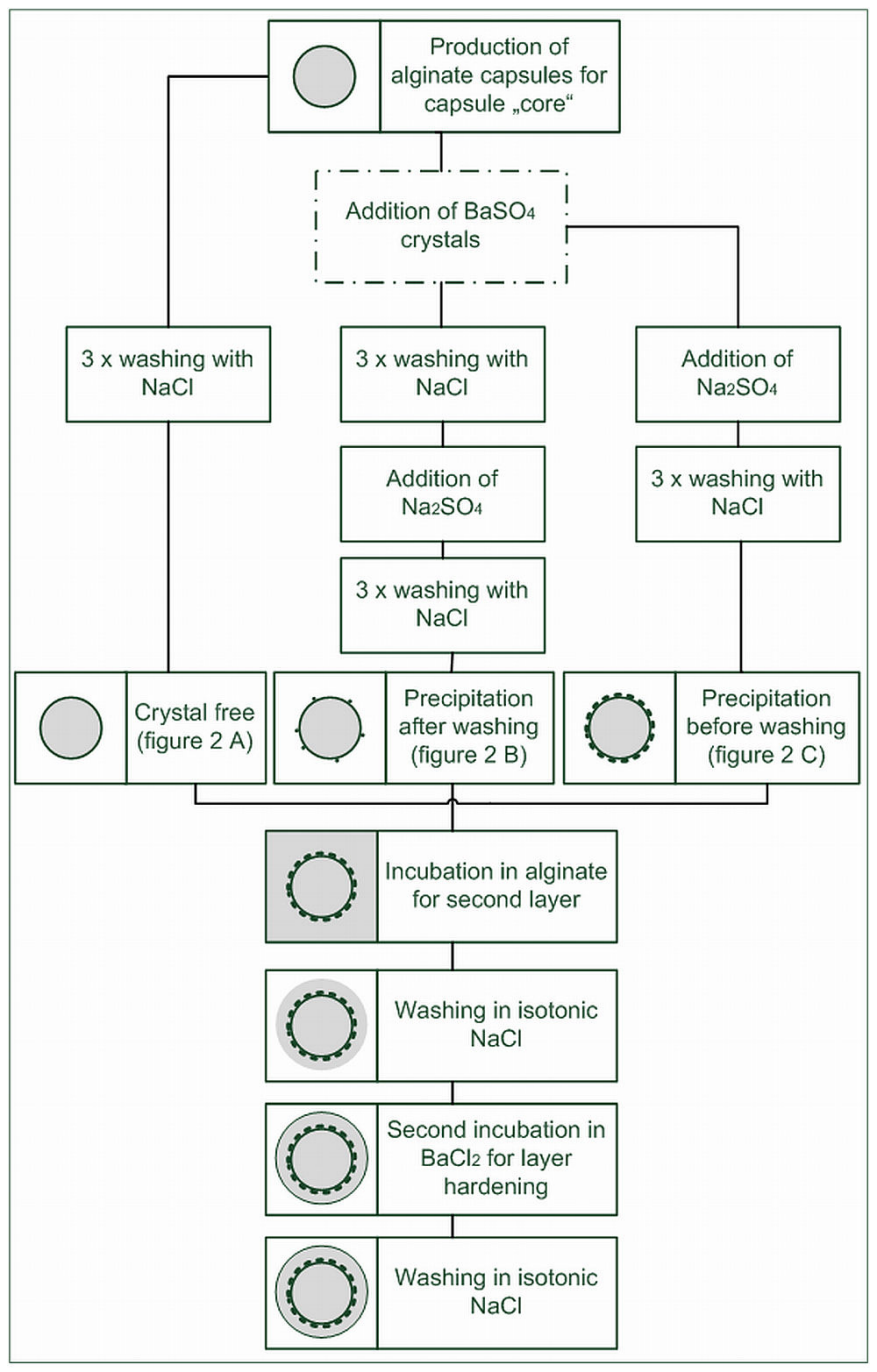
Flow chart for production of alginate bilayers with and without BaSO_4_ crystals.

Three different methods were used to apply alginate layers on core capsules (see also Figure 1). A: crystal free alginate layer application. Alginate core capsules were washed 3 times with isotonic NaCl to remove BaCl_2_ solution and pelleted. 500 µl of the pellet was transferred to alginate solution of different concentrations (w/v: 0.1%, 0.2%, 0.3%, 0.4%, 0.5%, 0.6% or 0.7%) and incubated for up to 10 min with gentle shaking. The incubation time was carefully controlled and varied for some experiments. Capsules were washed two or three times in NaCl to remove unbound alginate and suspended in 5 ml isotonic NaCl. 15 ml 20 mM BaCl_2_ solution (isotonic with NaCl) was added to harden the second layer and, after 20 min incubation, the capsules were finally washed with isotonic NaCl before transfer to culture medium or storage in isotonic NaCl. B: precipitation after washing. After incubation in BaCl_2_, capsules were washed 3 x in isotonic NaCl. 20 mM Na _2_SO_4_ solution (isotonic with NaCl) was added to precipitate unbound Ba^2+^. BaSO_4_ crystals can now be found on the capsule surface, creating a kind of Ba^2+^ deposit. 20 min later, the capsules were collected in a cell sieve with 70 µm mesh and washed three times with isotonic NaCl solution to remove unbound crystals. 500 µl of the capsule pellet were transferred to alginate solution and treated like described under A.C: precipitation before washing. An equal amount of 20 mM Na _2_SO_4_ solution was added at the end of incubation time of the core capsules. Precipitation of BaSO_4_ crystals was observed. After 20 min incubation, capsules were washed 3 x with isotonic NaCl, transferred in alginate solution and treated as described under A. Figure 1 demonstrates the production of layered alginate beads.

A fourth method was established during experiments for optimizing the insulin outcome of encapsulated Langerhans’ islets. Here, 3% HSA (human serum albumin) was added to alginate for production of core capsule and layer as well as in the polymerization solution and isotonic NaCl. After production of core capsule and washing in NaCl the layer was applied as following: The capsule pellet was taken up in a mix of 20 ml NaCl solution, 2.5 ml BaCl_2_ polymerization solution and 2.5 ml Na _2_SO_4_ solution to produce a fine layer of BaSO_4_ crystals. This solution was incubated for 3 min under constant gentle shaking. Capsules were washed by using a cell sieve and flushed with isotonic NaCl. About 500 µl capsule pellet were put in 0.65% alginate solution (+ 3% HSA) and incubated under gentle shaking for 6 min. Washing off the unbound alginate and polymerization of the alginate layer were performed as described above.

### Swelling stability of capsules

Capsules were transferred in tubes containing different media for swelling stability testing namely PBS (phosphate buffered saline, without Ca/Mg), cell culture medium (DMEM + 10% FBS) and pure FBS (fetal bovine serum). The tubes were kept at 37°C. Samples were taken and put into a petri dish which was treated with a small amount poly-L-lysine to avoid capsule movement during observation. Pictures were taken and total diameter of capsule and core capsule diameter were measured (software NIS-elements AR3.X, Nikon, Japan). The layer thickness was calculated by subtraction of core capsule diameter from capsule diameter.

### Statistical analysis

Each experiment was performed for 3 times. Mean values and standard deviation were calculated using standard excel (Microsoft Office 2007) or origin (OriginPro 8G) applications. Statistical significance was calculated using students t-test. Threshold for statistical significance was 5%.

## Results and Discussion

**Figure 2:**
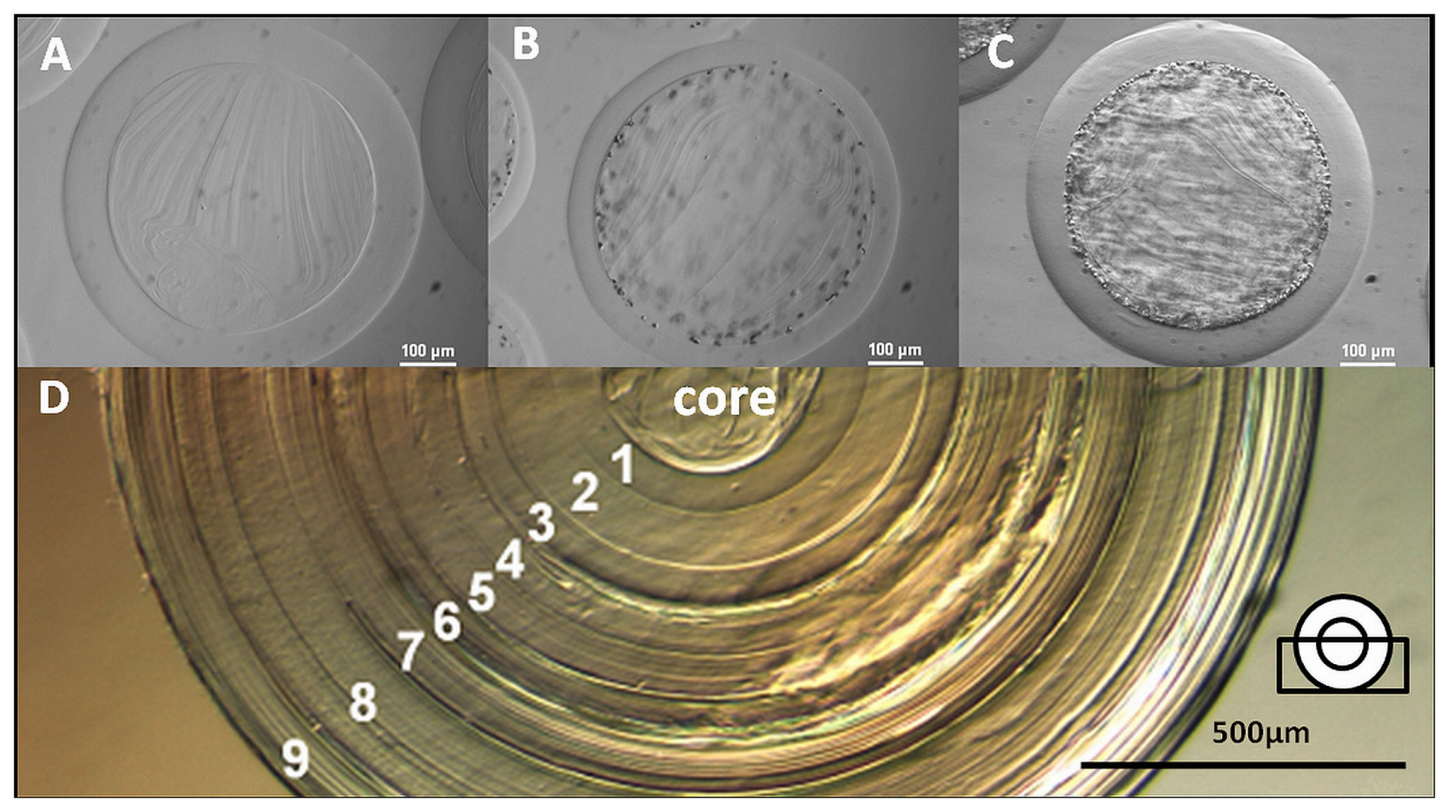
Phase contrast microscopy of alginate layers. **A**–**C**: Alginate bilayers after 10 minutes incubation in 0.3% alginate solution; the varied crystal density due to the different precipitation procedures causes a change in resulting mean layer thickness; **A**: crystal free; **B**: precipitation after washing with NaCl; **C**: precipitation before washing with NaCl; **D**: One core capsule with 9 step by step absorbed alginate layer (0.7%). Note a slight compression of the inner core capsule, which was possibly due to syneresis. The final size of this capsule system was about 2 mm.

Core capsules were always produced using 0.65% UHV alginate. An alginate layer was added according to the protocol using different methods for BaSO_4_ crystal application and different concentrations of alginate. Total diameter and core capsule diameter were measured to get the mean layer thickness. At least 40 capsules were measured to get the mean value and three independent experiments produced between 128 and 145 capsules per group. Use of the crystal gun during the production of the core capsules influenced the layer thickness in 5 of 8 groups significantly with a slight tendency that CG treatment produces thicker layers (table 1, Figure 2 A).

**Table 1:** Mean alginate layer thickness [µm] in dependence of different variables.

	**0.3%**		**0.5%**		**0.7%**	
	**Std**	**CG**	**Std**	**CG**	**Std**	**CG**
**0**	72.6 ± 8.3^#^	65.1 ± 13.4*	66.2 ± 11.8^#^	67.4 ± 7.3^#^	75.9 ± 8.3^#^	64.4 ± 7.6*
**+**	35.0 ± 6.9	31.6 ± 6.2*	35.9 ± 5.5	32.0 ± 4.5*	40.3 ± 4.8	33.6 ± 5.3*
**++**	64.8 ± 12.5	65.9 ± 14.9	61.9 ± 7.1	63.0 ± 5.9	65.5 ± 6.1	66.3 ± 8.8

Table 1 presents mean layer thickness of coated alginate capsules in dependence of alginate concentration, core capsule (0.65% alginate with crystal gun treatment (CG) or without (Std)) and BaSO_4_ crystals on the capsule surface (0 = crystal free, + = few crystals due to precipitation after NaCl washing or ++ = many crystals due to precipitation before NaCl washing). Total diameter and core capsule diameter were measured to get the mean layer thickness. At least 50 capsules were measured to get the mean value. Incubation time was 10 min. Note that precipitation after washing decreases the layer thickness significantly while any other factors do not influence the mean layer thickness of about 60-70 µm. * indicates the groups, in which CG treatment influenced significantly the layer thickness. **#** indicates the groups, in which second layers without BaSO_4_ precipitation (0) are significantly different from second layers with massive precipitation (++).

The thickness of the alginate layer was not dependent on the alginate concentration used for second layer formation (table 1). Concentrations of 0.2%-0.7% were tested and the layer thickness did not vary (for better overview only 0.3%, 0.5% and 0.7% were shown in table 1). Alginate solution containing 0.1% did not form a stable alginate layer and UHV alginate over 0.7% is difficult to handle due to the high viscosity.

Precipitation of an excess of BaSO_4_ crystals on the core capsule also did not result in a thicker alginate layer (table 1, Figure 2 C). The only treatment, which reduced the mean layer thickness significantly, was washing the core capsules with NaCl after production and addition of Na _2_SO_4_. This procedure produced a few tiny BaSO_4_ crystals on the surface of the core capsule and following incubation in alginate solution resulted in alginate layers which had only about half of the thickness (Figure 2 B).

Second layers without BaSO_4_ precipitation (Figure 2 A) and layers with strong precipitation (Figure 2 C) are very similar in values but the difference is in 4 of 6 cases significant. Second layers with precipitation after washing (Figure 2 B) are always significantly thinner compared to Figure 2 A and Figure 2 C.

Experimental staining of the core capsule with alcian blue before application of second layer (e.g. for better visibility of the core) produced an artifact which influenced the layer thickness in a non predictable way and was therefore abandoned. The second incubation in BaCl_2_ solution for polymerization of the layer was necessary to harden the layer. Incubation experiments in culture medium showed, that layers without second incubation were already from the point of production less visible, less contrasty and lost their integrity within a few days in contrast to the hardened layers.

Alginate layer addition was reproducible. We added up to 9 layers of alginate to one core capsule and found the layer thickness to increase slightly with every layer. The core capsule and the inner layers decreased slightly during the experiment, possibly due to syneresis of the gel layers while we observed the outer layers forming tree ring like structures. Application of alginate layer resulted generally in a slight decrease of the core capsule diameter of 50-60 µm (e.g. for 0.3% alginate layer). Although for application purpose only one alginate layer around a core capsule would be the most interesting method, multiple alginate layer deposition was possible with and without BaSO_4_ crystals. Using an alginate concentration of 0.3% resulted in more fragile layers and could not be extended to more than three layers. Figure 2 D shows nine absorbed layers using 0.7% alginate and even more layers are possible. The experiment was stopped because the capsule reached a diameter of 2 mm and was difficult to handle without damage. Multilayered capsules like this might be an interesting tool for controlled release studies or cell communications models.

**Figure 3:**
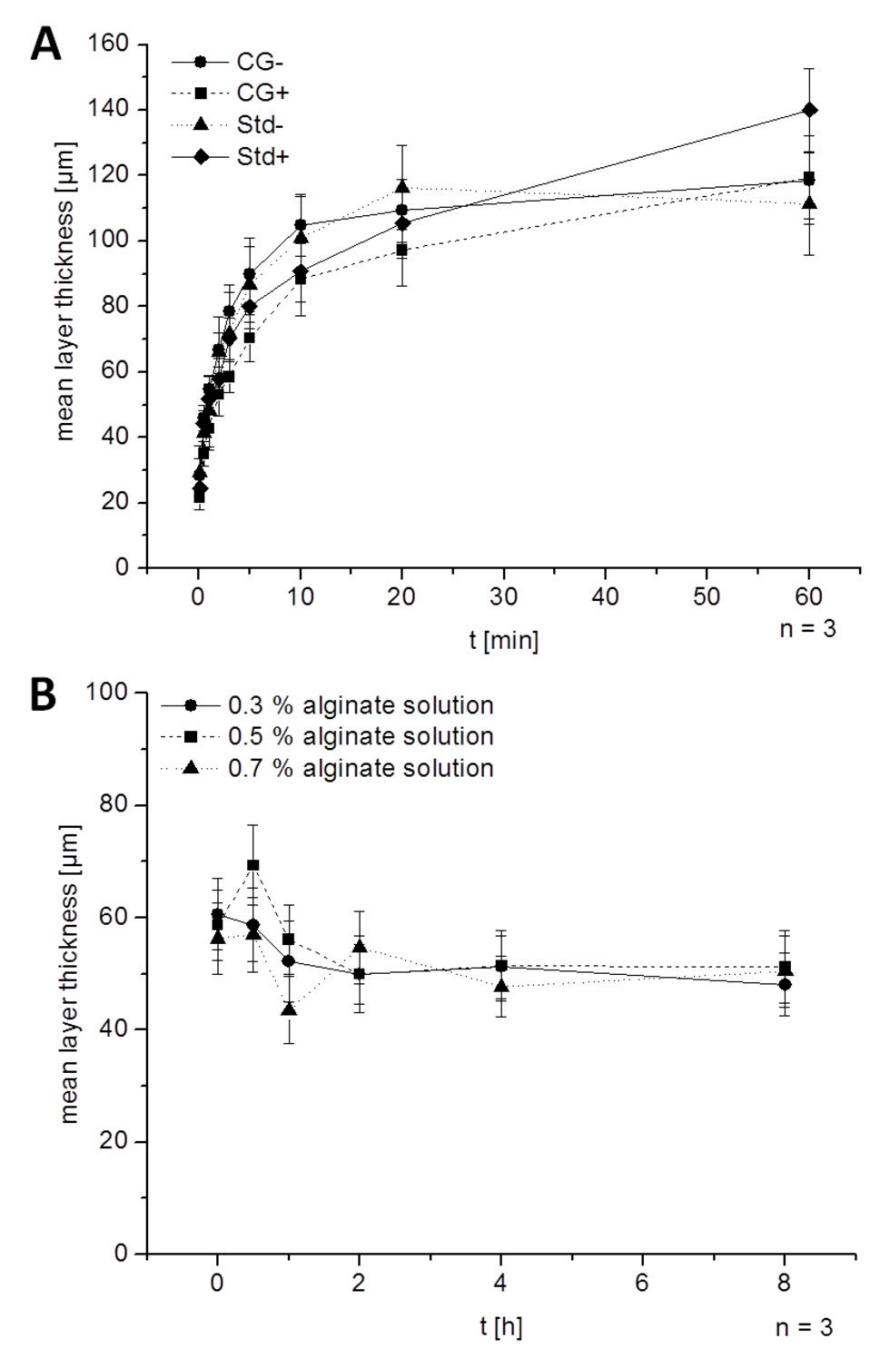
Mean alginate layer thickness. **A**: Thickness of alginate bilayer increases with incubation time in alginate. Results for incubation in 0.7 % alginate solution are shown; CG-: crystal gun core capsule without BaSO_4_ precipitation; CG+: crystal gun core capsule with BaSO_4_ precipitation; Std-: core capsule without crystal gun, without BaSO_4_ precipitation; Std+: core capsule without crystal gun, with BaSO_4_ precipitation **B**: The layer thickness varies if the capsules were coated directly after production (t = 0) or previously stored in isotonic NaCl. Mean layer thickness decreases slightly within the first two hours of storage, but stays subsequently stable up to 21 days (data not shown). Core capsules were produced out of 0.65% alginate solution without the crystal gun approach but with BaSO_4_ precipitation.

To examine the time dependency of the layer formation and the deposition mechanics, the incubation time in alginate was varied. The formation of the alginate layer starts when a core capsule hits the alginate solution and ends when the unbound alginate is washed away with NaCl solution. We found the layer formation to be a very fast process. Layer thickness increased rapidly during the first 5 minutes and almost reaches the maximum within 10 min (Figure 3 A). After 10 min there was a clear difference between BaSO_4_ crystal coated capsules and crystal free ones. The second alginate layer stopped growing on crystal free capsules at about 10 min while that on crystal coated capsules still increased.

Previously produced core capsules could be coated with a second alginate layer after storage in isotonic NaCl solution. The interval between core capsule production and double layer formation had no influence after the first hour. Directly after production the layer thickness was slightly higher and decreased within the first hour of storage (Figure 3 B). Afterwards the layer thickness was stable even after 21 days in isotonic NaCl (data not shown).

The time dependency of the alginate layer formation, as shown in Figure 3 A is a strong indicator for an adsorption reaction. Layer thickness increases very quickly in the first 10 min reaching saturation after 15 min. It is not clear whether the initial adsorption is caused by physisorption or chemisorption. Vorlop et al. presumed an excess of cross linking ions in gelled alginate [31] which could explain chemisorption of alginate monomers to the surface. The ability of alginate to bind more than one Ca^2+^ ion per 2 hydroxyl groups has been proven by Morris et al. 1978 [7] and Braccini et al. [8]. The 15 min incubation time in 20 mM BaCl_2_ solution may load the core capsule with an excess of cross linking ions which are more or less loosely bound to double or single hydroxyl groups of alginate, the so called half-egg-box binding [8]. However, an adsorption process purely caused by physical forces (physisorption) is reasonable and probable as it does not need the hypothesis of the presence of unbound crosslinking agents.

But, unbound or loosely bound Ba^2+^ could explain why we got an equal alginate layer with or without BaSO_4_ crystals. In both cases, there is a Ba^2+^ reservoir which attracts and binds the second layer. This hypothesis of ionic binding of the layer is supported by the observation that the second layer is even quite stable even without second incubation in Ba^2+^ solution but in contrast to the control it started dissolving within a few days in cell culture medium (data not shown).

Washing core capsules after production and precipitation of BaSO_4_ resulted in smaller and fewer crystals on the surface (see Figure 2 B) and the layer thickness of alginate was smaller than in all other experiments. In this case, we presume that the outer layer of the core capsule is poor in Ba^2+^. The few remaining BaSO_4_ crystals are less effective than the excess BaSO_4_ crystal layer, which was produced by precipitation before washing.

**Figure 4:**
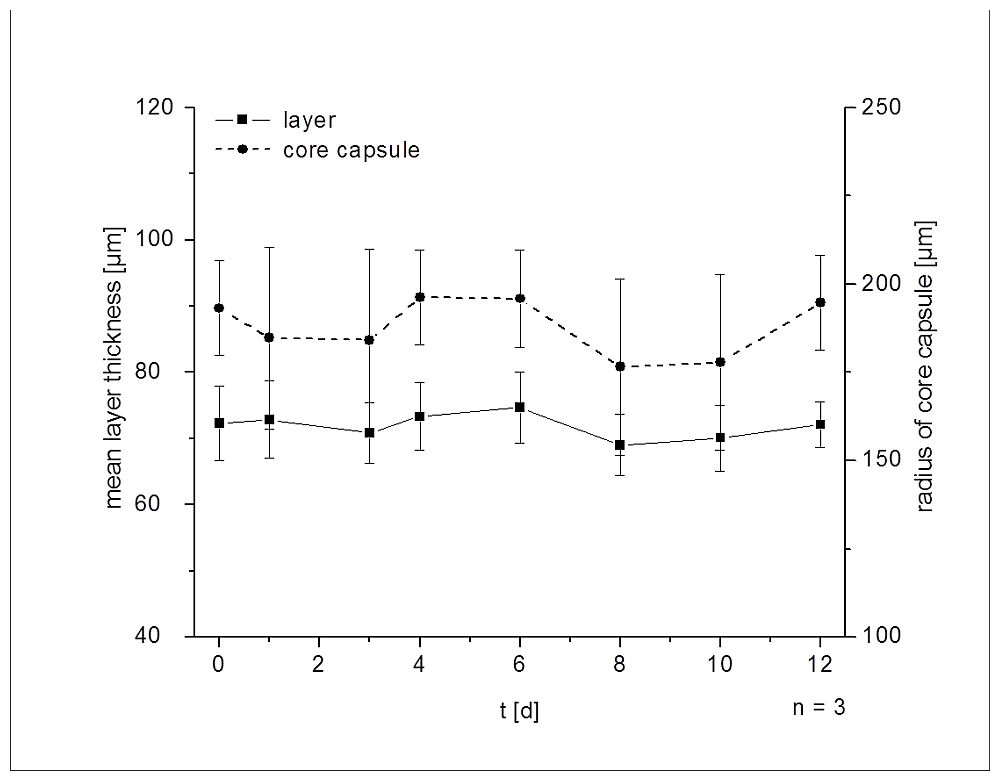
Swelling behavior of core capsules and alginate layers in DMEM based culture medium (containing 10% FBS); core capsules were produced without the crystal gun approach using 0.65% alginate (dashed lines); double layer capsules were produced as described using alginate solution with 0.7% (solid lines); capsules were fixated by poly-L-lysine coated 24 well plate to reduce movement during long term observation.

For investigation of long term stability alginate double layer capsules were kept at 37°C in different media: PBS (without Mg/Ca), culture medium (DMEM + 10% FBS) or pure FBS to watch swelling behavior. Figure 4 is representative for all tested media and for all tested alginate concentrations (0.3%, 0.4%, 0.5%, 0.6% and 0.7%). The size of the core capsule as well as the double layer was stable over 2 weeks. Fluctuations are more due to sampling of up to 50 different capsules.

The extraordinary *in vitro* and *in vivo* stability of UHV alginate, polymerized with barium has been shown before [9] and the second layer was found to be as stable as the core capsule. The stability of the second layer requires UHV alginate because experiments with commercially available alginate showed a disconnection, a clearly visible gap between the core and the second layer, regardless of the core material (data not shown). Possibly, this is due to the swelling mechanics of low viscosity alginate which are different to those of UHV alginate. The long term stability *in vitro* and *in vivo* of massive UHV Ba-alginate capsules is also due to its low swelling susceptibility.

**Figure 5:**
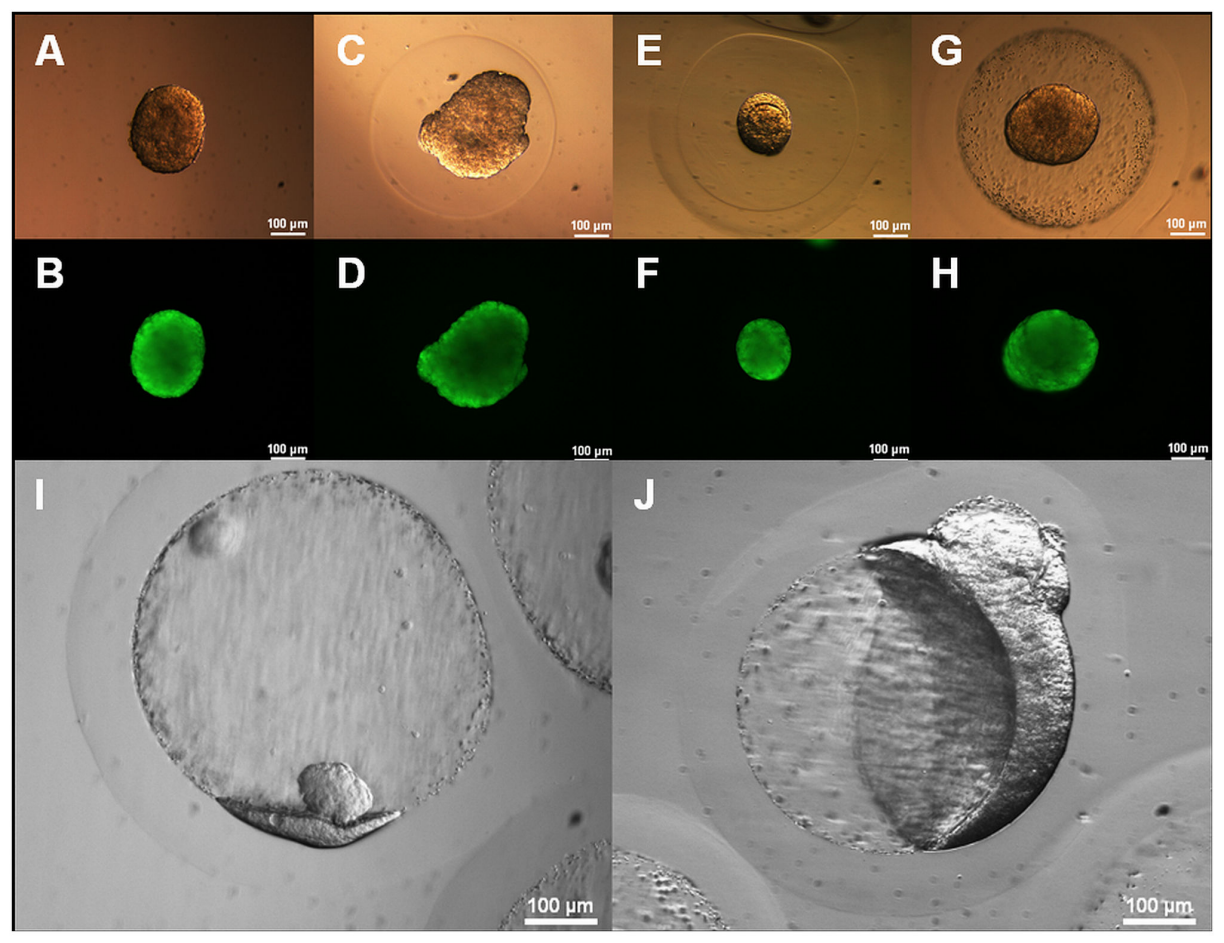
Examples of encapsulated RIN-m spheroids after 10 days of cultivation; Top row: transmission microscopy; Bottom row: fluorescence microscopy via live-dead assay (fluorescein diacetate and ethidium bromide); A/B: free RIN-m spheroid; C/D: standard encapsulated RIN-m spheroid; E/F:RIN-m spheroid encapsulated using the bilayer approach without BaSO_4_ precipitation; G/H:RIN-m spheroid encapsulated using the bilayer approach with BaSO_4_ precipitation. Due to cell growth spheroids grew out of core capsules and grew into the interface between the core capsules and the adsorbed alginate layers; **I**: early stage of interface growth of RIN-m spheroid; **J**: advanced stadium of interface growth after 14 days of cell culture the shape of the cell mass growing around the core capsule is clearly visible.

Viability of embedded cells was not affected by the extra alginate layer. Lethal diffusion inhibition was not observed. Rin-m (murine insulinoma cell line) spheroids were encapsulated according to the protocol (control without encapsulation, capsules without double layer respectively) and cultured for 10 days (Figure 5). Viability was constantly high (over 90%, detected after life dead staining by image analysis) in all samples.

Proliferating cells can break through an alginate capsule if they are immobilized near the surface. The double layer delays this breakout effectively as Figure 5 (I, J) shows. Culturing those capsules over a period of 14 days the cells were forced to grow between the core capsule and the alginate layer, forming finally a kind of large hollow spheroid. Further application of this technique for controlled production of e.g. multilayer spheroids might be an interesting point.

**Figure 6:**
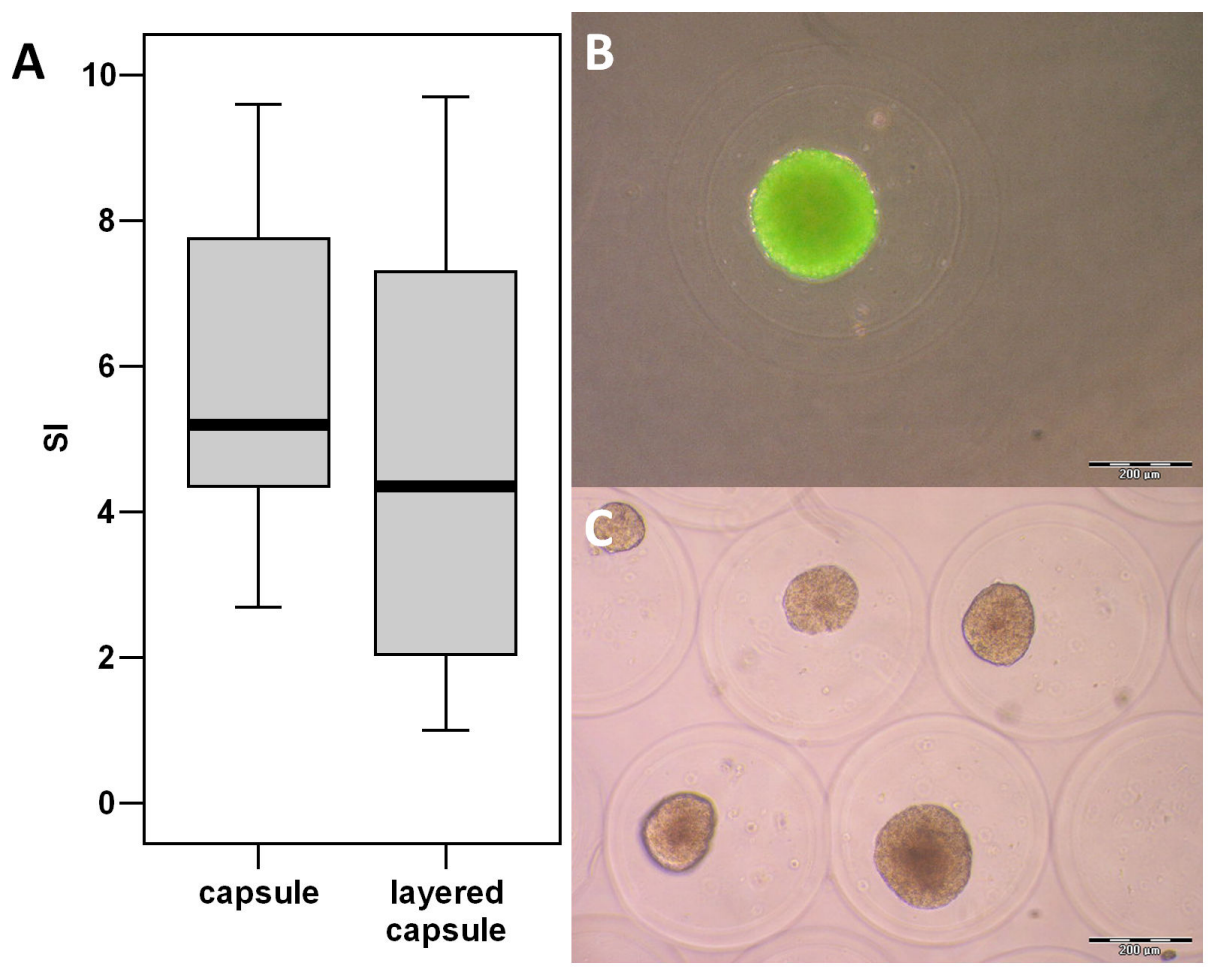
Encapsulated Langerhans’ islets. **A**: Insulin release of encapsulated islets with and without double layer expressed as stimulation index, p=0.16 (no significant difference), **B**: FDA/EB viability staining of encapsulated islet (green = vital), **C**: microscopic image of encapsulated islets with alginate double layer.

Murine Langerhans’ islets survived the double encapsulation well. In all performed experiment cells were still vital and able to produce insulin after 24 h of cultivation (Figure 6). Insulin stimulation indices therefore varied strongly due to the double encapsulation protocol, possibly due to a close-meshed alginate layer at the capsule surface or the mere increase of diffusion distance. After some optimization experiments, resulting in the fourth layer application protocol, the stimulation index increased just like encapsulated islets without double layer (Figure 6 A, no significant difference) but further research is necessary to keep up a reliable stimulation index.

## Conclusions

Finally, we can say that the polycation free multilayer technique with UHV alginate was successful in the first experiments and provides a great potential for clinical use. The second alginate layer did not stop cell growth but restrained the cells effectively within the capsule. An extra alginate layer is also highly interesting for immunoisolated transplantation as cells or tissue fragments are better protected even if they are immobilized at the outer rim of the capsule and the double layer is permeable for the therapeutic factor insulin. The advantages of a defined cell free alginate layer and the possibility to use higher cell concentrations clearly outweigh the slight size increase of the capsules.
